# Terrestrial laser scanning in forestry: Accuracy and efficiency in measuring individual tree parameters

**DOI:** 10.1371/journal.pone.0331126

**Published:** 2025-09-05

**Authors:** Zhangmai Li, Qinghua Qiao, Zibin Han, Xinyi Liu, Yueyang Wang, Hongzhao Tang, Lei Deng

**Affiliations:** 1 The College of Resource Environment and Tourism, Capital Normal University, Beijing, China; 2 Natural Resources Survey and Monitoring Research Centre, the Chinese Academy of Surveying and Mapping, Beijing, China; 3 Hebei Bureau of Geology and Mineral Resources Exploration, Shijiazhuang City, China; 4 The Land Satellite Remote Sensing Application Center, Ministry of Natural Resources, Beijing, China; Universidade Federal de Uberlandia, BRAZIL

## Abstract

With the growing global emphasis on forest resource monitoring, evaluating the accuracy of retrieving key individual tree parameters-such as tree position, tree height, and diameter at breast height (DBH)-using Terrestrial Laser Scanning (TLS) has become an important research focus. TLS has been widely applied in forest surveys due to its significant advantages in data acquisition efficiency and measurement precision. However, studies on the accuracy of extracting forest parameters from single-station, single-scan TLS data remain limited, underscoring the need for systematic evaluation and validation. This paper analyzes the accuracy and effectiveness of TLS in extracting structural parameters (tree height and DBH) and its position using *Poplar* and *Styphnolobium* as examples by using TLS, Airborne laser Scanning (ALS), and combining with field measurements. Results show that tree height estimates from single-scan TLS is limited in accuracy: the RMSE of 11.61 m in the *Populus* plot and 2.13 m in the *Styphnolobium* plot. Within a 50 m radius, single-scan TLS achieves a tree detection rate of 55.96–64.26% and a DBH RMSE of 1.60 cm (RRMSE: 9.03%). In addition, the point root mean square error of individual tree measurements remains at 0.11 m. These findings highlight the potential of TLS as an effective tool for forest inventory and provide a basis for evaluating the reliability of TLS-based plot measurements.

## 1. Introduction

Estimating forest biomass is essential for evaluating forest resources, ecosystem functionality, and sustainability. It also serves as a key indicator of ecosystem health [1]. Accurate monitoring of forest biomass is fundamental to maintaining ecosystem stability. It can inform strategies for climate change mitigation [2]. Traditional approaches often rely on fixed-area plots established in forested environments [3]. Biomass estimation is usually derived from measurements of tree height, diameter at breast height (DBH; 1.3 m above ground), and stand density, combined with species- or region-specific allometric equations [4]. However, manual acquisition of these parameters is labor-intensive and time-consuming, constraining its scalability for large-area assessments [5]. Terrestrial laser scanning (TLS) can provide high-resolution three-dimensional spatial data. When integrated with automated algorithms, it enables efficient and precise extraction of key parameters, including DBH, tree height, crown width, and tree position (i.e., the coordinates of the trunk cross-section center). This technology improves the efficiency and precision of forest parameter estimation [6]. In the last few years, TLS has been increasingly applied in forest monitoring and considered an important tool for forest structure assessment [7].

Recent advancements in LiDAR-based approaches have demonstrated the strong potential of TLS in retrieving individual tree parameters with high accuracy. Various algorithms have been proposed to improve DBH estimation from multi-scan TLS data. Specifically, a circular-elliptical fitting approach demonstrated a 4.7% reduction in mean error compared to traditional circular models [8]. Additionally, applying the Hough transform to merged multi-station TLS point clouds has further enhanced DBH retrieval accuracy in complex scanning environments [9]. Furthermore, Calders found that TLS-based tree height estimates strongly correlated with destructive sampling, achieving a correlation coefficient of 0.988 [10]. Notably, multi-scan TLS configurations have consistently outperformed conventional field-based methods in terms of both accuracy and efficiency [11]. At the stand level, evaluations of tree detection rates and height accuracy have confirmed the feasibility of TLS for operational forest inventories [12]. Montoya developed “treetool”, an open-source Python tool for automatic DBH extraction assessment, aiming to improve monitoring efficiency [13]. Despite these advancements, the use of TLS in forest still presents several technical challenges. Variations in terrain, dense understory vegetation and high stand density have been reported to adversely affect DBH retrieval accuracy using TLS, especially under heterogeneous conditions [14].

In summary, previous studies have investigated the capability of TLS in retrieving forest parameters and evaluating their associated accuracies. Most assume that multi-station TLS provides high retrieval accuracy. However, systematic evaluations of single-scan TLS performance remain limited, particularly with respect to the accuracy of tree height, DBH, and tree position measurements. This study aims to assess the feasibility of single-scan TLS in forest plot inventories, using *Poplar* and *Styphnolobium* as representative cases. By integrating datas from Unmanned Aerial Vehicle (UAV), TLS, and field measurements, we evaluate the accuracy of single-scan TLS in estimating individual tree parameters (tree height, individual tree position, and DBH). This study forms a foundation for assessing the reliability of TLS-derived plot measurements.

## 2. Materials and methods

### 2.1 Study area

**Fig 1:**
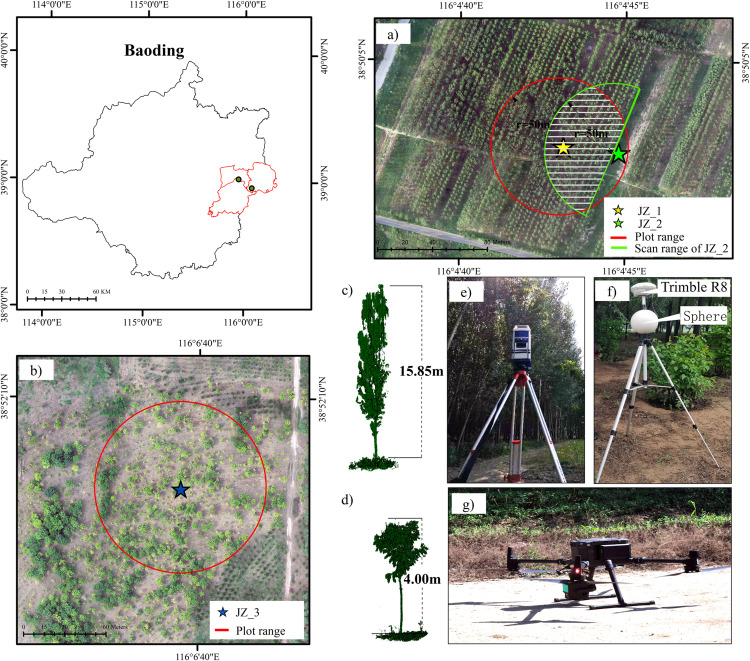
Study area (a) Plot 1 area (b) Plot 2 area (c) *Poplar* tree in Plot1 (d) *Styphnolobium* tree in Plot2 (e) Stonex X300 (f) Trimble R8 and Sphere (g) DJI M300 RTK multirotor UAV and L1 sensor.

The experimental area of this study is located in Xiong’an New Area, China. The area is located in the mid-northern latitude zone, with a warm temperate-type continental climate and four distinct seasons, with an average annual temperature of 11.9 °C and an average temperature of 26.1 °C in the hottest month of July. The altitude elevation ranges from 7 to 19 meters. The natural longitudinal slope is about one thousandth of a degree, with a relatively flat geographical position and no obvious topographic ups and downs. The study area contains two plots: Plot 1 (Fig 1a) and Plot 2 (Fig 1b), each a circular area with a 50-meter radius centered on stations 1 and 3(JZ_1,JZ_3), respectively. Plot 1 is a *Poplar* forest (Fig 1c). Plot 2 is *Styphnolobium* forest (Fig 1d). The weather was clear and windless at the time of data collection. The *Poplar* plot was densely wooded, with regularly spaced planting rows and a sparse understory of weeds and some shrubs. The *Styphnolobium* plot featured sparse tree cover, with the understory largely devoid of vegetation. An overview map of the study area is shown below (Fig 1).

### 2.2 Data acquisition and pre-processing

#### 2.2.1 Terrestrial laser scanning data.

The TLS equipment used in this study is the Stonex X300 instrument (Fig 1e), a 3D laser scanner that uses infrared Light Detection and Ranging (LiDAR) to extract forest sample parameters. The parameters of this instrument are shown in the Table 1.

**Table 1:** Specifications of laser scanning systems.

Technical specifications	TLS	ALS
Maximum distance range	300 m	450 m
DGNSS precision	H: ± 8 mm + 0.5 ppm V: ± 15 mm + 0.5 ppm	H: ± 10 mm + 1 ppm V: ± 15 mm + 1 ppm
Range systematic error	<4 mm@50 m	3 cm@100 m
Laser wavelength	905 nm	905 nm
Scanning speed	40000 pts/s	480,000 pts/s (multiple echoes)

TLS data was collected on July 20, 2023, under clear, windless conditions. Two single-station scans were performed for Sample Plot 1 (Fig 1a). The first scan (JZ_1), covering a 360-degree horizontal range, is indicated by the yellow star in Fig 1. The second scan (JZ_2), conducted in the opposite direction, had a horizontal range of 180 degrees and was parallel to the boundary of the *poplar* forest (Fig 1a). Within a 50-meter range, the scanning area of JZ_2 overlapped approximately 50% of the total area of Sample Plot 1.

One scan of sample plot 2 (see Fig 1b) as a horizontal 360-degree scan (blue star in Fig 1). All three TLS scans are in fine mode. The target sphere position is placed at a position without object obstruction around 10m from the TLS base station. The positions of the target sphere (Fig 1f) and the base station were measured using a Trimble R8 (Qianxun RTK, H: ± 8 mm + 0.5 ppm, V ± 15 mm + 0.5 ppm) to obtain their coordinate data. The measured coordinates of the base station and the target sphere were used to calibrate the TLS point cloud data. The point cloud densities of JZ_1, JZ_2, and JZ_3 were obtained at 3063 pts/m^2^, 2392 pts/m^2^, and 2291 pts/m^2^, respectively.

#### 2.2.2 Airborne lidar system and RGB data.

UAV data collection of plots was accomplished using a DJI M300 RTK multi-rotor UAV (Fig 1g) equipped with an L1 sensor. The ALS has a built-in RTK module. The DJI L1 sensor is composed of LiDAR and RGB sensors, and it has the ability to acquire LiDAR data and RGB images at the same time. The flight time is up to 55 minutes, which ensures sufficient data collection time. During the flight, the UAV flew at an altitude of 100 m. The flight speed was 4 m/s.

The ALS data was collected with 80% laser bypass overlap and point cloud densities ranging from 919–1677 pts/m^2^.In Sample Plot 1, 374 trees were extracted from the ALS data, with 295 trees identified by JZ_1 and 225 by JZ_2. In Sample Plot 2, 251 trees were extracted from the ALS data, and 217 trees were identified by JZ_3 (Table 2).

**Table 2:** Sample plot information.

Plot	1	2
Species	*Poplar*	*Styphnolobium*
Average height(m)	15.85	4.00
Average DBH(cm)	15.44	17.72
Number of trees observed by TLS	295	217
Number of trees observed by ALS	374	251

When taking RGB images, the heading overlap and side overlap rate were set to 70% and 84%, respectively. The RGB data was then processed; The RGB orthoimages of the sample plots were generated through multi-image stitching, achieving a resolution of 2.7 cm.

### 2.3 Tree height

The main steps of extracting tree height data from the TLS point cloud data after calibration include: de-coarsening, Digital Elevation Model (DEM) generation, point cloud normalization, single-tree segmentation, and information extraction. The above process was completed in Point Cloud Automata (PCA v4.3). Tree height extraction from ALS was processed using the same method as TLS point cloud data. Because of the high accuracy of the tree height extracted by the ALS [15], the tree height extracted from the UAV point cloud was used as a reference value to analyze the ability of the TLS to measure the tree height of the sample plots.

The test of tree height accuracy was carried out in Plot 1 and Plot 2, respectively.We utilized Spatial Join methods to align the data from the two stations. First of all, using the sample plot individual tree results extracted from the TLS (JZ_1 and JZ_3) and ALS data, respectively, and analyzing them based on their spatial relationship, i.e., if the distance of the individual tree measured by the two is less than 1.5 m, they are initially considered to be the same tree, and then the RGB image is used for visual discrimination to achieve the matching between the same individual tree of TLS and ALS [16]. After matching sample plot 1 and sample plot 2 had 223 and 154 trees screened, respectively (see Table 2). Finally, an accuracy analysis is conducted.

### 2.4 The detection rate

The method for extracting individual tree count from TLS data is the same as that for tree height extraction. The number of individual trees visually interpreted from UAV RGB imagery served as a reference for calculating the tree detection rate.

To evaluate the data acquisition efficiency of a single TLS scan, we used the tree detection rate as an indicator. Specifically, within the overlapping area (50 m radius) of two base stations, the actual number of individual trees was counted based on high-resolution RGB images, and the number of trees detected by TLS at each base station in this area was recorded.The detection rate is calculated according to the formula. The formula is shown in Equation 1.


$$\text{Treedetectionrate}=\frac{treedetectionfromTLS}{treeobservedfromRGMmap}\times 100\%\mathrm{\backslash ]}$$ (1)

In Plot 1, an experiment was conducted within the overlapping area of the 50-meter range from two single scans.

### 2.5 Diameter at breast height

DBH data was extracted from the calibrated TLS point clouds using the same processing method as tree height retrieval. The DBH of the tree closer to one of the TLS stations was used as the reference value. Based on the measurement capability of the TLS device in this experiment, i.e., a measurement error of 4 mm within 50 m (Table 1), we analyzed the DBH extraction capability of the TLS using measurements centered on the base station and within a 50 m radius. Firstly, the data obtained from the two TLS stations (JZ_1 and JZ_2) were matched using a spatial join method. Then, the subtraction method was utilized to get the difference in the diameter at breast height of the same tree measured by the two stations. Based on the DBH matching results from the two stations, the DBH of trees closer to one of stations served as the reference value, while those farther from the station were used as the measurement values. The individual tree with the least stem obstruction near each base station was selected to form a dataset of measurement and reference values, and the filtered dataset includes the parameters of 12 individual trees. Finally, an accuracy analysis is conducted.

### 2.6 Tree position

Tree position data was extracted from TLS point clouds using the same method as tree height and DBH. Then, we analyzed the accuracy of TLS measurement positions using the location data of one station as a reference value.

Since it is difficult to accurately measure the geographic coordinates of each tree in the forest with GNSS technology, we used the following approach to evaluate the accuracy of single-tree position using measurements from Sample Plot 1: First, extracting the position parameters of individual trees from the point clouds calibrated from JZ_1 and JZ_2, respectively. Subsequently, the Spatial Join method was employed to identify trees that were co-matched to the TLS at both stations within the 50-meter measurement range, resulting in a total of 91 individual trees. Finally, we assessed the accuracy of single-scan TLS measurement positions using the location data of a selected station as a reference.

### 2.7 Evaluation metrics

The accuracy of TLS in estimating forest tree height, detection rate parameters, DBH and tree position, was evaluated using root mean square error (RMSE), relative root mean square error (RRMSE), mean error (ME) and mean absolute error (MAE). The formulas for RMSE, RRMSE, ME, MAE are shown in Equations (2–5).


$$\text{RMSE}=\sqrt{\frac{\sum_{i=1}^{n}{\left({\text{X}}_{\text{est,i}}–{\text{X}}_{\text{ben,i}}\right)}^{2}}{\text{n}}}$$ (2)

$$\text{RRMSE}=\frac{RMSE}{{\overset{―}{\text{X}}}_{\text{ben}}}*100\%\mathrm{\backslash ]}$$ (3)

$$\begin{array}{c} \text{ME}=\frac{\text{1}}{\text{n}}\sum {\mathrm{\backslash nolimits}}_{\text{i}=\text{1}}^{\text{n}}\left({\text{X}}_{\text{est,i}}–{\text{X}}_{\text{ben,i}}\right) \end{array}$$ (4)

$$\begin{array}{c} \text{MAE}=\frac{\text{1}}{\text{n}}\sum {\mathrm{\backslash nolimits}}_{\text{i}=\text{1}}^{\text{n}}\left|({\text{X}}_{\text{est,i}}–{\text{X}}_{\text{ben,i}})\right| \end{array}$$ (5)

In the formula, X_est,i_ is the measured value, X_ben,i_ is the reference value, ‾x is the average of the reference values and n is the number of trees.

The accuracy of the TLS position measurements was evaluated in terms of the root mean square error of the point. The formula for the root mean square error of a point is shown in Equation (6)


$$\text{M}=\sqrt{\frac{\sum_{i=1}^{n}\left(\Delta {\text{X}}_{i}^{2}+\Delta {\text{Y}}_{i}^{2}\right)}{\text{n}}}$$ (6)

M is the point root mean square error (m), ∆x_i_ and ∆y_i_ are the differences in the coordinates of the detected points (m), and n is the total number of coordinate points, i.e., the number of individual trees.

## 3. Results

### 3.1 Accuracy of tree height

**Fig 2:**
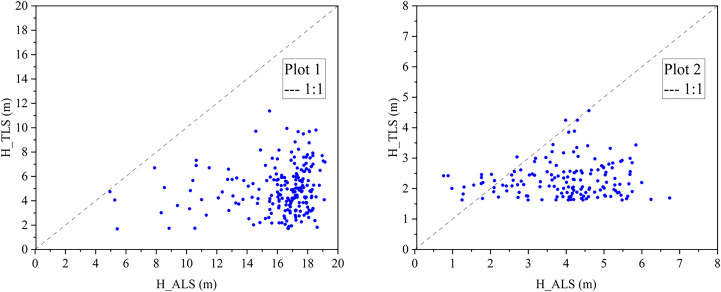
H_ALS and H_TLS discrete point.

Based on the tree heights extracted by ALS (H_ALS, X-axis) and TLS (H_TLS, Y-axis), Scatter plots of the height values measured by ALS and TLS in the two sample plots were obtained, as shown in Fig 2.

As illustrated in Fig 2, tree height estimates derived from TLS in Plot 1 are systematically lower than those from ALS, with all points falling below the 1:1 reference line. A similar pattern is observed in Plot 2, indicating a underestimation trend by TLS across both plots. A quantitative accuracy assessment is conducted by comparing TLS-derived tree heights with ALS data, as summarized in Table 3.

**Table 3:** Accuracy analysis of tree height measured by TLS.

	RMSE (m)	RRMSE (%)	ME (m)	MAE (m)
JZ_1	11.61	71.60	−11.22	11.22
JZ_3	2.13	53.32	−1.69	1.82

**Fig 3:**
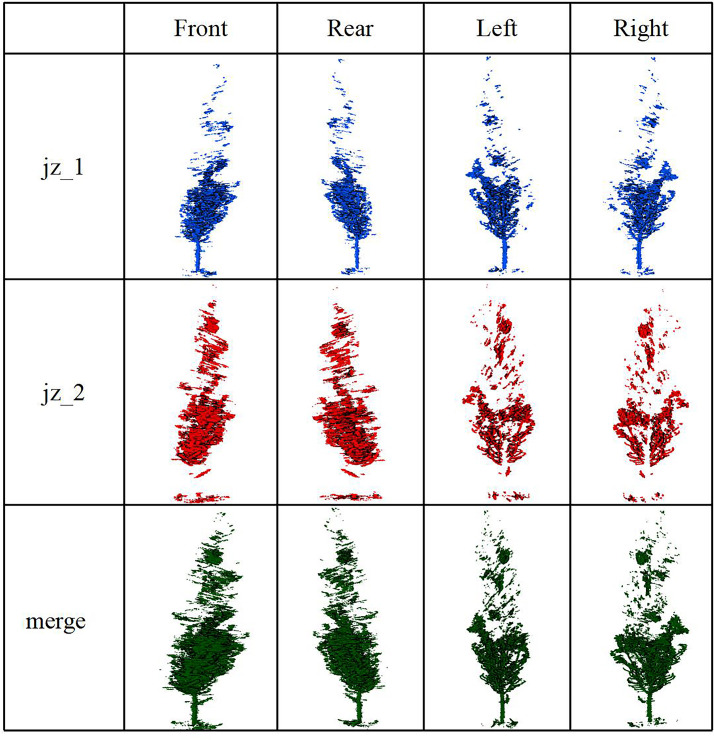
Comparison of single wood point clouds for TLS scanning.

As shown in Table 3, the tree height in the *Poplar* plot exhibits the largest deviation, with an RMSE of 11.61 m and a RRMSE of 71.60%. In the *Styphnolobium* plot, TLS still underestimate height but with reduced error (RMSE = 2.13 m; RRMSE = 53.32%). Additionally, we obtained the point cloud result after merging JZ_2 with JZ_1, as shown in Fig 3.

It can be seen from the above figure that the integration of two TLS scans from different viewpoints can improve crown delineation and trunk visibility, suggesting enhanced point cloud completeness and potential in tree height estimation accuracy.

All in all, these findings confirm that single-scan TLS is inadequate for accurate tree height estimation, underscoring the necessity of multi-viewpoint integration or supplemental data for accurate canopy characterization.

### 3.2 Accuracy of detection rate and DBH

A total of 277 trees were identified by manual interpretation of the RGB images in the overlapping area. The number of trees detected by each TLS scan, as well as the combined detection outcome, is summarized in Table 4 along with corresponding detection rates.

**Table 4:** Tree detection rates derived from TLS.

Base station	Number of detected trees	Total number of trees	Tree detection rate(%)
JZ_1	178	277	64.26
JZ_2	155	277	55.96
JZ_1 + JZ_2	218	277	78.70

**Fig 4:**
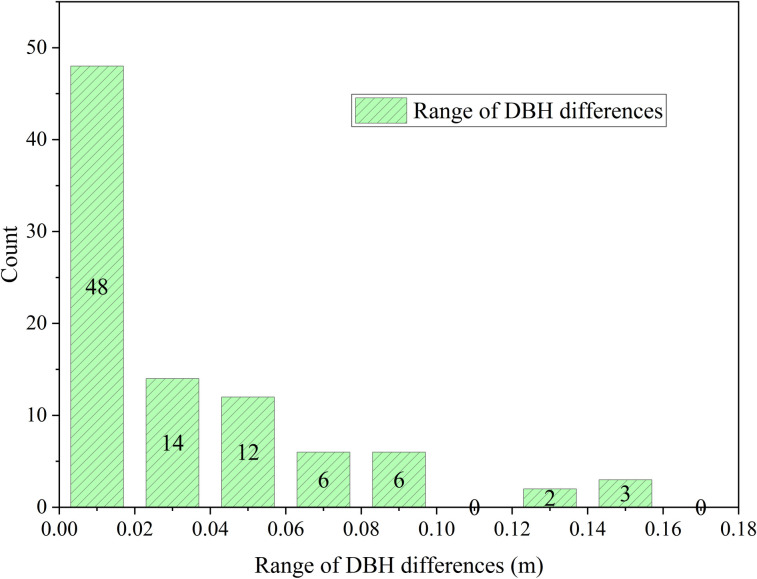
Statistical graph of breast diameter difference range between two base stations.

According to the above table, single-scan TLS achieves tree detection rates ranging from 55.96% to 64.26%, while dual-station integration increase the rate to 78.70%, indicating improvement with additional viewpoints. These findings suggest that single-station TLS is capable of detecting a substantial proportion of individual trees, and dual-station integration can further enhance the detection rate, although the overall improvement remains limited. The difference in DBH can be calculated to obtain Fig 4.

Based on the above (Fig 4), 90.24% of DBH differences between the two TLS base stations fall within the 0–6 cm range at a 50 m radius. An accuracy assessment of DBH measurements from TLS reveals an RMSE of 16.20 mm and an RRMSE of 9.03%, indicating high measurement accuracy. Additionally, ME of –11.80 mm and MAE of 15.50 mm are observed, further confirming the reliability of single-scan TLS in capturing DBH under the experimental conditions.

### 3.3 Accuracy of individual tree position

**Fig 5:**
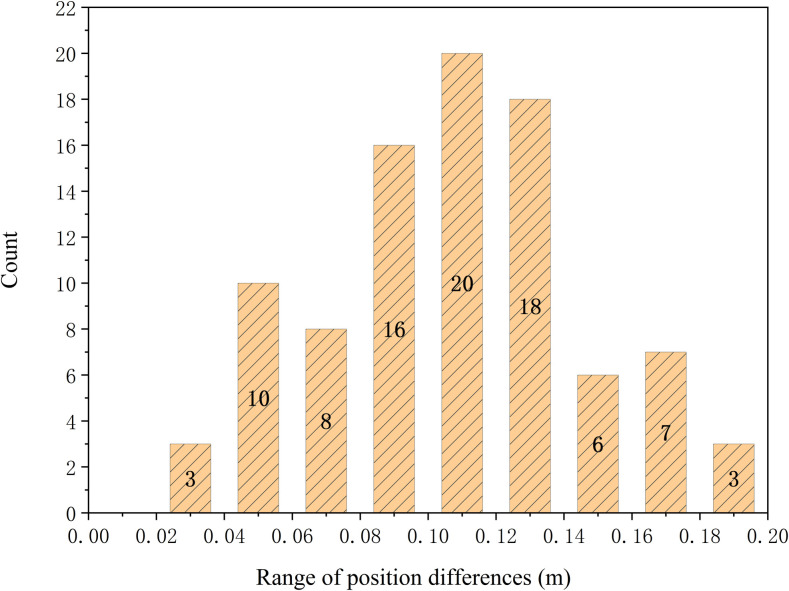
Position difference quantity statistics.

A histogram of planar position differences was generated from the 91 co-identified individual trees within the 50 m overlapping area scanned by JZ_1 and JZ_2 (Fig 5).

**Fig 6:**
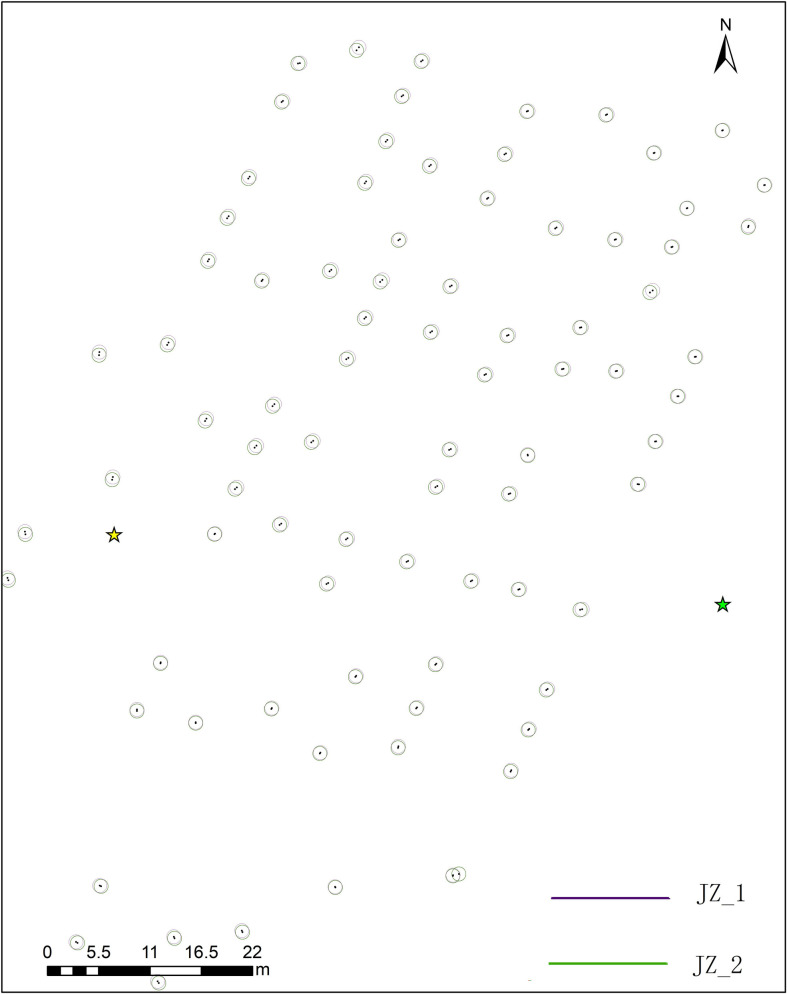
Vector distribution of partial tree trunk positions within plot 1.

As is evident from Fig 5, the differences between position are mainly centered between 8 cm and 14 cm. Subsequently, vector maps of trunk positions within the sample plot are obtained based on the locations of individual trees scanned at both sites (Fig 6).

Fig 6 shows spatial discrepancies between corresponding tree locations derived from the two single-scan TLS datasets. The point root mean square error between matched tree centers from the two TLS scans is 0.11 m, indicating a high degree of geospatial accuracy achievable with single-scan TLS.

## 4. Discussion

### 4.1 Differences in tree height

Li used nine single-station multi-angle methods in their experiment to collect receipts from mixed forests within an artificial experimental area to investigate the ability of TLS to measure tree heights. Their experimental area was a rectangle of 6 × 6 or 12 × 12 trees, and they obtained a better fitting relationship between the measured heights and the heights obtained by TLS [17], contrary to the results we obtained. He did not fully take into account the complexity of the forest sample plots, conducting the study only in rectangular areas with only 36 and 144 trees and setting up a high number of stations. However, in real forest environments, the amount of data to be collected is larger and having multiple sites in a dense forest may not be easily achievable. The experiment in this study was conducted in a forest field sample plot rather than an ideal artificial experimental area. The results indicate that the tree height measurements obtained using single-station TLS in the sample plot have a relatively large error.

The *poplar* trees is high, and the proximity between the TLS and the trees limited the scanning line of sight. As a result, the crown tops of nearby trees were not captured. The complete crowns of distant trees could not be fully scanned due to occlusion. Because the *Styphnolobium* tree plots are relatively low and sparse, the error is relatively small. Consequently, the measurements were underestimated, leading to poor accuracy, as shown in Table 3. When using a single TLS to measure tree height in a forest sample plot, the distance between the TLS and the target object must be carefully considered. Additionally, point cloud data scanned by JZ_2 is registered and merged with JZ_1 using feature points, resulting in a more complete tree crown point cloud after processing (Fig 3). After aligning and merging the two stations, the tree heights measured by TLS become more accurate:the accuracy improved from 11.61 meters (single station) to 2.37 meters. However, multi-station TLS measurements can reduce the efficiency of forest resource surveys.

With the advancement of LiDAR and UAV technologies, The combination of LiDAR and UAV has become the mainstream method for measuring tree height in forests. Quan et al. demonstrated that UAV-based LiDAR can achieve tree height extraction accuracy of up to 0.5785 m [18], offering advantages in extracting individual tree counts while providing high precision in estimating canopy structural parameters. For extracting tree height parameters across an entire sample plot, Airborne LiDAR (ALS) offers greater efficiency.

### 4.2 Evaluation for detection rate and DBH

The column density in plot 1 was 586 trees/ha, which is not a very high single station get rate at this forest density. The detection rate for single stations is relatively low, ranging from 56% to 64%. Even when combining the results from two stations observed from different directions, the overall acquisition rate is only 80% (see Table 4). Because of the distribution characteristics of the dense vegetation, the occlusion effect between trees is more pronounced when data is collected from two angles, resulting in relatively low retrieval rates. Conducting two scans not only increased data collection time and workload, but also failed to meet the requirements of traditional forest sample plot surveys(all trees), as the total number of trees is a key parameter in forest resource assessment [19]. Although both JZ_1 and JZ_2 are single-station measurements, there is a difference in the angle at which they are measured, which leads to a significant difference in the effectiveness of their data acquisition. Therefore, careful consideration needs to be given to the selection of TLS locations. Sometimes, in order to ensure the best results, we may need to conduct site surveys with the help of high-resolution satellite images or drones to clearly locate the best placement of TLS base stations. Such careful consideration and preliminary investigation can help improve the accuracy and efficiency of TLS data collection.

In Sample Plot 1, the number of individual trees visually interpreted from UAV-acquired RGB images (Table 2) was significantly higher than that obtained from TLS,which could also be due to tree obstructions within the dense plot. Since plot 2 is relatively sparse, the number of individual trees extracted from ALS data is closer to that obtained from TLS. With continuous advancements in remote sensing technology, The integration of TLS and ALS in sample plot surveys is gradually maturing. We can geographically align the point cloud data scanned by TLS with those scanned by ALS in the sample plots. Matching between individual trees is then performed after parameter extraction. Based on the DBH measured by TLS and tree height and crown diameter (CD) measured by ALS, we can establish a functional relationship. Through this function, the diameter at breast height of the individual trees not measured by TLS can be calculated based on the ALS data [20], so as to obtain the single-tree parameters of the sample plots and even the whole region, and also save the survey Time. However, in some forest samples with more complex canopy conditions, the use of algorithms to segment ALS data into individual trees may result in wrong or missing segments. Therefore, it is necessary to improve the accuracy of the segmentation algorithm when applying this method in universal samples. This is to ensure that individual trees can be accurately identified and segmented in a variety of forest environments. Such improvements will help to increase the reliability and accuracy of the overall sample plot survey [21].

In addition, the reason for choosing to use the DBH closer to the base station as the reference value is twofold: firstly, from the point of view of equipment performance, the results of DBH measured in close proximity are more accurate. Secondly, the DBH obtained from manual measurement as the reference value itself has certain errors, such as the error related to the height of the ground and the reading accuracy of the DBH ruler may affect the accuracy of the measurement. In contrast, the advantage of extracting the diameter of the breast diameter in the software is that the same standard is used for each tree, for example, the algorithm for extracting the diameter of the breast diameter is unified, and the size of the diameter of the DBH is calculated at a height of 1.3 meters, which makes the results of computer processing more objective and accurate. To a certain extent, its measurements can be used as a kind of ground validation data [22]. Therefore, the DBH close to the base station is used as a reference value for the TLS’s DBH accuracy validation. At the same time Reddy analyzed the ability of TLS to measure the diameter of the chest in the sample plots with an average diameter of 16–19 cm, and he got the result that the accuracy of the measurement at a single station was 4.1 cm (RMSE), and the accuracy obtained from the experiments in this paper was even higher than that obtained by him [23]. After counting, it was found that the difference in diameter at breast height on both sides of the two base stations could reach up to 16 cm in some cases, but only accounted for 1.2% of the total. Despite the existence of the above phenomenon, such a result is in line with reality, there may be a certain degree of shading between trees, resulting in the lack of trunk point cloud data [24]. Analysis of the merged point cloud from dual-station scanning showed that the parameter extraction accuracy (RMSE = 13.85 mm) improved only slightly. This may be due to errors introduced during point cloud merging, affecting overall accuracy. In comparison, single-station TLS achieved similar accuracy with higher efficiency.

### 4.3 Position and equipment performance

When using TLS to collect tree position data from different directions within the same plot, the positional error for the same tree should theoretically be within the error range of the RTK instrument. However, in the actual experiment, the distance between the same tree measured at two different locations exceeded the instrument’s error range. The possible reasons for this discrepancy can be attributed to two factors: first, errors in coordinate measurements introduced additional deviations during point cloud calibration; second, occlusion between tree trunks led to incomplete point cloud data, affecting the algorithm’s calculation of tree positions. As technology continues to advance, new algorithms are expected to further enhance the accuracy of individual tree position extraction. Devices with auto-leveling and built-in GPS functions have been developed. These new devices not only save measurement time but also reduce relative position errors between the target (base station/target sphere) and GNSS, minimizing the impact of human error on forest position parameter extraction. The application of advanced algorithms and equipment significantly improves operational convenience while providing higher measurement accuracy and efficiency in forest plot surveys.

The TLS equipment used in this study exhibits a systematic error of less than 4 mm over a 50-meter range. Other TLS devices available on the market offer higher measurement accuracy and extended range, enabling more precise forest parameter estimation and greater measurement efficiency. [25]. This study employed the Stonex X300, which has certain limitations. Technological advances in the accuracy and performance of the equipment continue to improve, the Leica ScanStation P40/P50, for example, in 270 meters within the system error of 1.2 mm + 10 ppm, in more than 1,000 meters when its error is 3 mm + 10 ppm.Enhancements in the accuracy of TLS equipment may improve its precision in measuring forest parameters as well as improve the detection of trees. In addition, the equipment used in the paper requires RTK support for point cloud data acquisition, however, it is more difficult to achieve orientation in densely forested areas due to insufficient signal coverage. In recent years, handheld LiDAR has been developing rapidly, because handheld LiDAR utilizes Simultaneous Localization and Mapping(SLAM) technology and relies on its own sensors to obtain accurate high-frequency positioning information without relying strongly on Global Navigation Satellite System (GNSS), which is easy to use [26]. And the LiDAR using SLAM algorithm can operate in the dense understory, but its walking rule requires closed-loop is difficult to realize in some environments, so its accuracy is not guaranteed in complex environments. Cabo found TLS to be more reliable and competitive in extracting the ground surface of forest sample plots by comparing it with handheld mobile laser scanning [27].

## 5. Conclusions

This paper analyzes the capability of TLS single-scan measurements in forest sample analysis by studying the extracted positions, tree heights and DBH of individual trees. The study achieved significant results, particularly in estimating the positional accuracy and DBH of individual trees. TLS demonstrated good performance and satisfactory results in extracting the number of individual trees. The accuracy of TLS single-scan measurements for tree DBH and positions meets the requirements for sample plot collection. To a certain extent, TLS technology improves the efficiency of forest resource investigation, and produces more satisfactory results when combined with ALS data: TLS and ALS can be combined to obtain the tree parameters of the whole sample plot, or even the forest parameters of the whole region under certain conditions. In summary, TLS provides high accuracy and effectiveness in measuring the tree location and DBH, as well as extracting the number of individual trees in a forest sample, but is not suitable for measuring tree height in a plot. However, only one TLS device was used for data collection in this experiment, and thus the possible differences between different devices were not considered. In addition, the forest environment in which the experimental area is located is relatively simple, and the effects of factors such as terrain undulation, differences in forest density, and understory vegetation on the degree of TLS shading were not fully considered, which limits the comprehensiveness of the study to a certain extent.

## Supporting information

S1 Data Raw measurement data. (XLSX)

## References

[1] Lu D. The potential and challenge of remote sensing‐based biomass estimation. Int J Remote Sens. 2006;27(7):1297–328. doi: 10.1080/01431160500486732

[2] Kumar L, Mutanga O. Remote sensing of above-ground biomass. Remote Sens. 2017;9(9):935. doi: 10.3390/rs9090935

[3] Liang X, Kankare V, Hyyppä J, Wang Y, Kukko A, Haggrén H, et al. Terrestrial laser scanning in forest inventories. ISPRS J Photogramm Remote Sens. 2016;115:63–77. doi: 10.1016/j.isprsjprs.2016.01.006

[4] Schumacher FX, Hall FDS. Logarithmic expression of timber-tree volume. J Agric Res. Beltsville: U S Dept Agriculture, Agricultural Research Service; 1933 Dec;47:0719–0734.

[5] Liang X, Hyyppä J, Kaartinen H, Lehtomäki M, Pyörälä J, Pfeifer N, et al. International benchmarking of terrestrial laser scanning approaches for forest inventories. ISPRS J Photogram Remote Sens. 2018;144:137–79. doi: 10.1016/j.isprsjprs.2018.06.021

[6] An A, Chen M, Zhao L, Zhu H, Tang F. Density Adaptive Plane Segmentation from Long-Range Terrestrial Laser Scanning Data. IGARSS 2022 - 2022 IEEE International Geoscience and Remote Sensing Symposium [Internet]. Kuala Lumpur, Malaysia: IEEE; 2022 [cited 2024 Feb 19]. p. 7511–7514. Available from: https://ieeexplore.ieee.org/document/9884779/

[7] Liu J. Stem detection from terrestrial laser scanning data [Master]. Chongqing Jiaotong University. 2023.

[8] Wang Pei. Adaptive estimation method for diameter at breast height based on terrestrial laser ScanningBu Guochao. Laser Optoelectron Prog. 2016;53(8):082803. doi: 10.3788/lop53.082803

[9] Liu L, Pang Y, Li Z. Individual tree DBH and height estimation using terrestrial laser scanning (TLS) in a subtropical forest. Linye Kexue/Scientia Silvae Sinicae. 2016;52:26–37.

[10] Calders K, Newnham G, Burt A, Murphy S, Raumonen P, Herold M, et al. Nondestructive estimates of above‐ground biomass using terrestrial laser scanning. Methods Ecol Evol. 2014;6(2):198–208. doi: 10.1111/2041-210x.12301

[11] Zhou J, Zhou G, Wei H, Zhang X. Estimation of the Plot-Level Forest Parameters from Terrestrial Laser Scanning Data. IGARSS 2018 - 2018 IEEE International Geoscience and Remote Sensing Symposium [Internet]. Valencia: IEEE; 2018 [cited 2024 Jan 30]. p. 9014–9017. Available from: https://ieeexplore.ieee.org/document/8518529/

[12] Yrttimaa T, Saarinen N, Kankare V, Hynynen J, Huuskonen S, Holopainen M, et al. Performance of terrestrial laser scanning to characterize managed Scots pine (Pinus sylvestris L.) stands is dependent on forest structural variation. ISPRS J Photogram Remote Sens. 2020;168:277–87. doi: 10.1016/j.isprsjprs.2020.08.017

[13] Montoya O, Icasio-Hernández O, Salas J. TreeTool: a tool for detecting trees and estimating their DBH using forest point clouds. SoftwareX. 2021;16:100889.

[14] Liu G, Wang J, Dong P, Chen Y, Liu Z. Estimating individual tree height and Diameter at Breast Height (DBH) from Terrestrial Laser Scanning (TLS) data at plot level. Forests. 2018;9(7):398. doi: 10.3390/f9070398

[15] Giannetti F, Puletti N, Quatrini V, Travaglini D, Bottalico F, Corona P, et al. Integrating terrestrial and airborne laser scanning for the assessment of single-tree attributes in Mediterranean forest stands. Eur J Remote Sens. 2018;51(1):795–807. doi: 10.1080/22797254.2018.1482733

[16] Wu Y, Mao Z, Guo L, Li C, Deng L. Forest volume estimation method based on allometric growth model and multisource remote sensing data. IEEE J Sel Top Appl Earth Observations Remote Sens. 2023;16:8900–12. doi: 10.1109/jstars.2023.3313251

[17] Li Y, Hess C, von Wehrden H, Härdtle W, von Oheimb G. Assessing tree dendrometrics in young regenerating plantations using terrestrial laser scanning. Ann For Sci. 2014;71(4):453–62. doi: 10.1007/s13595-014-0358-4

[18] Quan Y, Ming Z, Zhen Z, H Y. Modeling crown characteristic attributes and profile of Larix olgensis using UAV-borne LiDAR. J Northeast For Univ. 2019;47(11):52–8. doi: 10.13759/j.cnki.dlxb.2019.11.011

[19] Hu X. Research on tree counts extraction from UAV imagery based on fusion watershed algorithm [Master]. Northeast Forestry University. 2021.

[20] Guo L, Wu Y, Deng L, Hou P, Zhai J, Chen Y. A Feature-level point cloud fusion method for timber volume of forest stands estimation. Remote Sens. 2023;15(12):2995. doi: 10.3390/rs15122995

[21] Zhu C. Deep learning individual tree segmentation method for coupling airborne LiDAR and digital orthophoto map [Master]. Changchun Institute of Technology. 2022.

[22] Dan L, Pang. A review of TLS application in forest parameters retrieving. World For Res. 2012.

[23] Suraj Reddy R, Rakesh A, Jha CS, Rajan KS. Automatic estimation of tree stem attributes using terrestrial laser scanning in central Indian dry deciduous forests. Curr Sci. 2018;114(01):201. doi: 10.18520/cs/v114/i01/201-206

[24] Wu J. Research on data acquisition and processing method for roadside tree based on in-vehicle 3D laser scanning. Urban Geotechn Invest Surv. 2018;(6):70–5.

[25] Wardius Y, Hein S. Terrestrial laser scanning vs. manual methods for assessing complex forest stand structure: a comparative analysis on plenter forests. Eur J Forest Res [Internet]. 2024 Jan 12 [cited 2024 Feb 1]; Available from: https://link.springer.com/10.1007/s10342-023-01641-1

[26] Zhao C. Handheld multi-sensor fusion slam algorithm research [Master]. Nanjing University of Information Science and Technology. 2023.

[27] Cabo C, Del Pozo S, Rodríguez-Gonzálvez P, Ordóñez C, González-Aguilera D. Comparing Terrestrial Laser Scanning (TLS) and Wearable Laser Scanning (WLS) for individual tree modeling at plot level. Remote Sens. 2018;10(4):540. doi: 10.3390/rs10040540

